# A Legionnaires’ disease outbreak associated with cooling towers in Warstein, Germany, August 2013

**DOI:** 10.1186/2047-2994-4-S1-P227

**Published:** 2015-06-16

**Authors:** A Jurke, A Maisa, F Renken, A Brockmann, C Lück, M Exner, I Daniels-Haardt

**Affiliations:** 1 Infectiology and hygiene, NRW Centre of Health, Münster, Germany; 2 Public Health Department, Soest, Germany; 3 Institute for Medical Microbiology and Hygiene, National Consulting Laboratory for Legionella, Dresden, Germany; 4 Institute for Hygiene and Public Health, University of Bonn, Bonn, Germany

## Introduction

Legionnaires’ disease (LD) results mainly from inhalation of aerosols containing the bacterium *Legionella pneumophila*, which may cause atypical severe pneumonia. Between the 1^st^ of August and 6^th^ of September an unusual cluster of patients with LD of unknown etiology in Warstein, North Rhine-Westphalia, Germany was notified to the public health authorities.

## Methods

Laboratory investigation was performed according to requirements of European case definition. A questionnaire had been used to narrow down possible sources of infection. A case-control study was conducted to identify possible sources of infection. Cases and controls were matched for age-group and sex. Odds ratio (OR), 95% confidence interval (CI) and p-values were calculated by logistic regression. Values of p<0.05 were considered statistically significant.

## Results

The outbreak accounted for 159 suspected and 78 laboratory-confirmed cases including one death. *Legionella pneumophila*, serogroup 1, subtype Knoxville, sequence type 345, could be identified as the epidemic strain. Cases were 19 to 94 years old, 64% were males. The case fatality rate was 1.28%, 91% of cases were hospitalised, 17% of those needed intensive care. In univariable analysis cases were almost five times more likely to smoke than controls (OR 4.81; 95% CI 2.33-9.93; p<0.0001). Furthermore cases were twice as likely to live within a 3 km distance from one identified infection source as controls (OR 2.14; 95% CI 1.09-4.20; p<0.027).

## Conclusion

This is the largest outbreak of LD in Germany to date. Due to a series of uncommon events, this outbreak was most likely caused by multiple sources involving industrial cooling towers. Quick epidemiological assessment, source tracing and shutting down potential sources as well as rapid laboratory testing and early treatment are necessary to reduce morbidity and mortality. Maintenance of cooling towers must be reliable to prevent such LD-outbreaks in the future

## Disclosure of interest

None declared.

